# Smile aesthetics in Pakistani population: dentist preferences and perceptions of anterior teeth proportion and harmony

**DOI:** 10.1186/s12903-024-04100-4

**Published:** 2024-03-29

**Authors:** Rizwan Jouhar, Naseer Ahmed, Muhammad Adeel Ahmed, Muhammad Faheemuddin, Seyed Ali Mosaddad, Artak Heboyan

**Affiliations:** 1 Department of Restorative Dental Sciences, College of Dentistry, King Faisal University, Al-Ahsa, 31982 Saudi Arabia; 2 Department of Prosthodontics, Altamash Institute of Dental Medicine, Karachi, 75500 Pakistan; 3 Department of Prosthodontics and Implantology, College of Dentistry, King Faisal University, Al-Ahsa, 31982 Saudi Arabia; 4 Department of Research Analytics, Saveetha Institute of Medical and Technical Sciences, Saveetha Dental College and Hospitals, Saveetha University, Chennai, India; 5 Student Research Committee, School of Dentistry, Shiraz University of Medical Sciences, Shiraz, Iran; 6 Department of Prosthodontics, School of Dentistry, Tehran University of Medical Sciences, North Karegar St, Tehran, Iran; 7 Department of Prosthodontics, Faculty of Stomatology, Yerevan State Medical University after Mkhitar Heratsi, Str. Koryun 2, Yerevan, 0025 Armenia

**Keywords:** Aesthetic smile, Dental photographs, Dental casts, Peston proportion, Golden proportion, Golden percentage, RED proportion

## Abstract

**Background:**

This study aimed to evaluate dentist perceptions of attractive smiles in the Pakistani population, considering different dental proportions.

**Methods:**

Maxillary casts and digital images were used to create symmetrical representations of anterior teeth. dentists’ preferences for good and bad teeth proportions, width/height ratios, and various dental proportions (golden, recurring esthetic dental (RED), golden percentage, Preston, and local/observed) were assessed using one sample and paired t-test. The Chi-square test was used to determine the gender disparities and factors affecting smile attractiveness. A *p*-value of ≤ 0.05 was taken as significant.

**Results:**

The RED proportion emerged as the preferred choice for normal-sized teeth, with specialists and general dentists favoring it over the golden proportion. For tall teeth, the golden proportion was predominantly preferred. The golden percentage received limited preference for aesthetic smile construction.

**Conclusions:**

The smiles created using the principles of RED proportion were opted as the most attractive by local dentists. Factors such as tooth arrangement, color, and midline were highlighted as essential considerations in aesthetic smile construction.

**Supplementary Information:**

The online version contains supplementary material available at 10.1186/s12903-024-04100-4.

## Introduction

Aesthetics in dentistry is one of the major concerns, especially for patients. When an individual smiles, how their smile is perceived plays a vital role in their self-confidence. Although what is considered an aesthetic smile varies from the perception of every different individual, the ideal standard for an aesthetic smile has not been established as such [1]. Different disciplines of dentistry play essential roles in enhancing the aesthetics of the patients, such as orthodontists modifying hard and soft tissues, prosthodontists replacing lost oral structures with artificial teeth, and restorative dentists restoring decayed and stained teeth [2]. All these efforts are made so the patients can meet their demands for the aesthetics they seek. In the past few decades, many different materials and techniques have been introduced to produce attractive outcomes in terms of dental aesthetics for patients [3]. The size and form of the maxillary anterior teeth play an essential role in the smile of the patients.

Several different tooth proportion theories have played an essential role in determining the corresponding widths of the maxillary anterior teeth. The golden proportion is one of the tooth proportions that states the relationship between mathematics and beauty [4]. According to the golden proportion, when applied to the smile design, the width of the maxillary anterior teeth should exist according to it [4]. The width of the maxillary lateral incisor should be 62% of the width of the maxillary central incisors, and the maxillary canine’s width should be 62% of the width of the maxillary lateral incisors. The golden proportion does not consider the body proportions, clinical crown lengths, and individuals’ body types [5]. Although the golden proportion is studied thoroughly, it is not always found in different ethnicities.

Factors such as body, face, and teeth should be considered in calculating the recurring esthetic dental (RED) proportion to overcome the deficiencies of the golden proportion. According to the RED proportion, the widths of the maxillary anterior teeth successively should remain constant as they advance distally [6]. When smiles are designed according to this principle, the width of the successive teeth, as viewed from the front, diminishes [7]. The width of the maxillary lateral incisors is reduced by a selected percentage compared to maxillary central incisors, and the width of maxillary canines is reduced by a percentage compared to maxillary lateral incisors. Generally, it has been noted that a 70% RED proportion has been recommended [8]. According to the 70% RED proportion, the width of maxillary lateral incisors has to be 70% of the width of maxillary central incisors, and the width of maxillary canine will be 70% of the width of maxillary lateral incisors [8]. Different studies have used maxillary anterior teeth height to analyze it with different tooth proportions. Some dentists have favored the RED proportion when considering the height of maxillary teeth [9].

The golden percentage, proposed by SR. Snow [10], offers a method for achieving aesthetically pleasing smiles through proportional tooth widths. It dictates that the central incisor should be 25%, the lateral incisor 15%, and the canine 10% of the total frontal width. The assigned values of 1.6, 1, and 0.62, respectively, contribute to the golden “percentage,” highlighting the dominance of central incisors. While studies show minor variations, Murthy and Ramani suggest mean values of 21.9–22.3% for centrals, 15.3–15.5% for laterals, and 12.0-12.6% for canines. Ethnic differences may account for variations, prompting considerations for population-specific adjustments [7]. Ali Fayyad’s [11] study on Arab students recommends values of 23%, 15%, and 12% for centrals, laterals, and canines, respectively, emphasizing the importance of ethnicity in applying the golden percentage theory to smile design.

The Preston proportion, introduced by Jack D. Preston [12], outlines an ideal relationship between the widths of maxillary anterior teeth to enhance smile aesthetics. It suggests that the lateral incisor should be approximately 66% of the central incisor’s width and the canine about 84% of the lateral incisor’s width. However, this guideline is not rigid, as natural dentition measurements vary, and individual aesthetics, ethnicity, and unique smile characteristics must be considered [13]. While the Preston proportion serves as a useful starting point for dental professionals, customization and a broader understanding of esthetic factors are crucial for achieving optimal results in restorations and cosmetic procedures.

When the maxillary central incisors are longer, to consider them aesthetically acceptable, they must also be wider to maintain the height-to-width ratio [13]. This produces larger and more dominant central incisors. Similarly, teeth that are short in length should be short in width for aesthetic purposes. In the local Pakistani population, very few studies have been carried out that evaluate dentists’ preference for aesthetic smiles using different tooth proportions. Furthermore, dentists in the local population sometimes do not consider using tooth proportions to produce an aesthetic smile according to the patient’s preferences, which may lead to unaesthetic results that can bother the patients and the dentists. This study aimed to evaluate dentists’ perceptions regarding attractive smiles in the local Pakistani population, considering the harmony between tooth proportions and maxillary anterior teeth height and width ratios. Furthermore, we also assessed the perception of attractive smiles amongst the general dentists and specialists.

## Materials and methods

### Study design and sample size estimation

This cross-sectional analytical study was conducted at Altamash Institute of Dental Medicine, Pakistan. The study duration was stretched from 2019 to 2021 due to the pandemic and the hustle of collecting clinical data. A nonprobability, convenience sampling technique was adopted to recruit participants in the study. For the first phase of the study Determination of crown width: height ratio of maxillary anterior teeth. The sample size was calculated using the public service of Creative Research Systems survey software, considering (62%) [5] prevalence of dental proportion. The estimated sample size at a 5% margin of error and 95% confidence interval was *n* = 230 participants with intact natural maxillary anterior teeth. The sample size for the 2nd phase perception of smile attractiveness, considering a 16.5% [9] response rate on smile attractiveness. The estimated sample size at a 5% margin of error and 95% confidence interval was *n* = 143 dentists after considering the 1,000,000 population.

### Ethical approval and participant consent

The ethical approval was acquired from the AIDM ethical review committee (AIDM/EC/06/2019/06). All participants signed the informed consent form.

### Subject criteria of the study

#### Phase 1

**Inclusion Criteria**:

- Subjects with intact six natural maxillary anterior teeth.
- Well-aligned teeth with class 1 incisor relationship.
- Age between 18 and 30 years at the time of examination.
- Absence of interdental spacing in maxillary teeth.
- No history of orthodontic treatment and prosthetic restoration in anterior teeth.
- No history of congenital conditions or trauma affecting facial form and appearance.

**Exclusion Criteria**:

- Missing any maxillary anterior teeth.
- Mal-apposed or malformed maxillary anterior teeth.
- Severe attrition and fractured anterior teeth.
- Caries/restorations in any of six maxillary anterior teeth.
- Gingival inflammation, hypertrophy, or periodontal disease.
- Facial asymmetry.
- Fractured dental models, including broken cast teeth, plaster wear, or casting defects.
- Photographs with poor resolution, blurriness, or unclear landmarks of teeth and face at 200x magnification.

#### Phase 2

**Inclusion Criteria**:

- Inclusion of dental practitioners.
- Participants from the Pakistani population across three generations identified by a national identity card (NIC).

**Exclusion Criteria**:

- Subjects with visual impairment, including eyesight weakness or blurred vision.
- Color blindness.
- Eye infections such as German measles or allergic conjunctivitis.
- Eye blindness.

### Dental impression and plaster cast making

Two hundred and thirty maxillary casts were fabricated using a perforated stainless-steel tray covering the hamular notches and fovea palatine. The tray was selected precisely to ensure a consistent 3–4 millimeters space for the impression material (Fast setting alginate hydrogum, Zhermack SpA, Badia Polesine, Italy). The borders of the tray were extended up to the functional sulcus depth, ensuring a thorough impression without causing any physical discomfort to the subjects. Subsequently, the impressions were meticulously poured using Type IV dental stone (ISO Type 3, Elite Rock Zhermack SpA, Badia Polesine, Italy) to achieve accurate and detailed maxillary casts.

### Measurement and analysis of anterior teeth

This investigation employed a digital camera body (Canon EOS, DSLR Camera, CMOS, 18 MP, 1920 × 1080p/30fps). The camera included an 18–55 mm + 75–300 mm built-in magnification lens to record sharp, precise, and repeatable photos. The collected 230 photos were reviewed for any anomalies, like malalignment, spacing, distortion, blurring, and unclear features, according to the inclusion criteria.

**Fig. 1:**
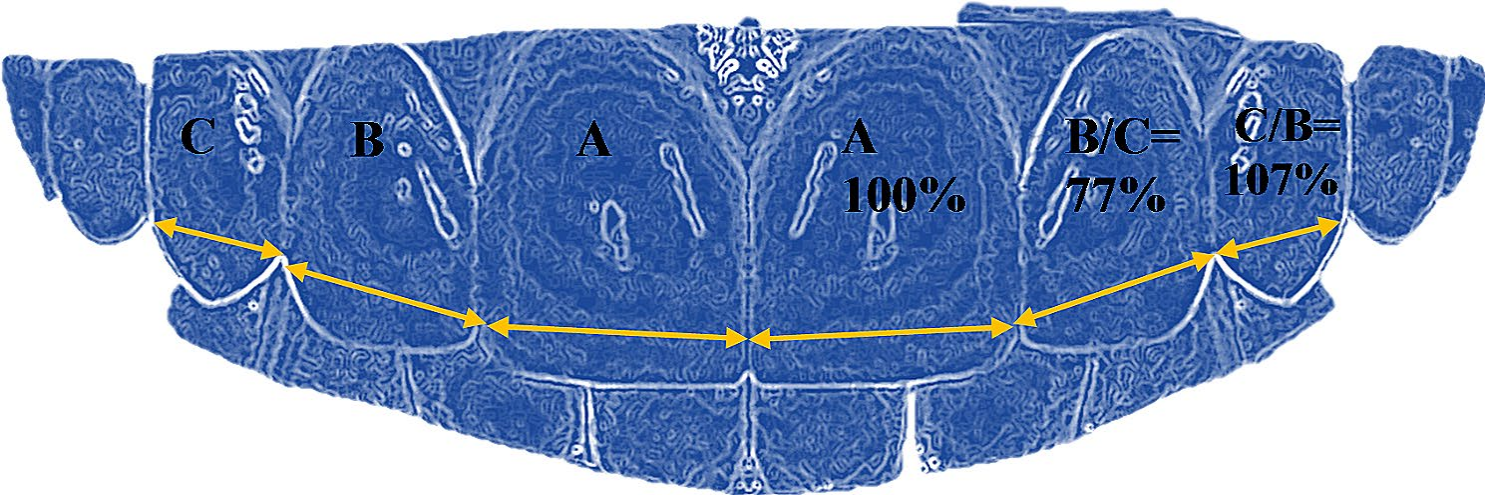
The proposed dental proportion values obtained in this study for the Pakistani population (Local/observed proportion)

**Fig. 2:**
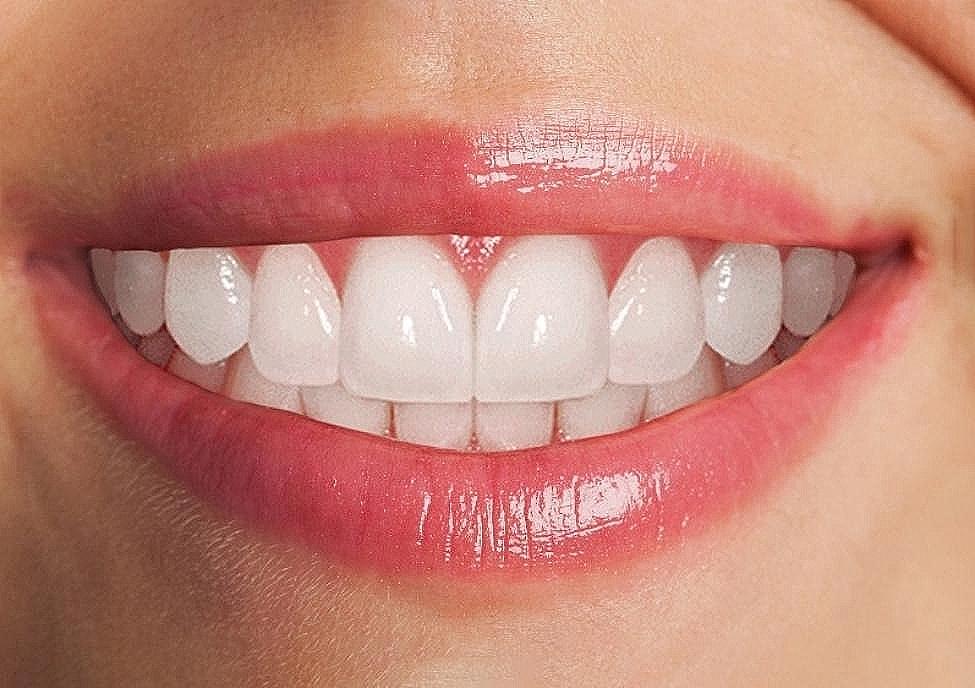
The reference smile to create other dental proportion images

The mesiodistal perceived width of anterior teeth from the included images was measured between the contact points with Adobe Photoshop software (version 21.0.2, San Jose, CA, United States). The actual width of anterior teeth was measured with a vernier caliper on the dental cast. Similarly, the length of teeth was measured from the cervical to the incisal edges of teeth at the middle one-third from the labial side. The width-to-height ratio of anterior teeth was calculated by dividing tooth width by the height recorded. The local or observed proportion of anterior teeth in the study was found by dividing the width of the lateral incisor tooth from the central incisor. Similarly, the canine width was divided by the adjacent lateral incisor tooth on each side of the arch, and the subsequent value was multiplied by 100 to achieve the percentage values. The mean dental proportion values were obtained (76.89% rounded to 77%) for right and left lateral incisor to central incisor teeth. Meanwhile, for the right and left lateral incisor and canine teeth (106.30% rounded to 107%), As shown in Fig. 1. To minimize photographic errors, the actual width of the maxillary anterior teeth obtained from dental casts was divided by the photographic width from pictures to calculate a conversion factor (W.H. Ward) [14]. The photographic teeth widths were then multiplied by this conversion factor to overcome magnification errors and achieve the true width captured in the photograph (Fig. 2). The mesiodistal teeth dimension obtained after assessing photographic error estimation was named the perceived width in this study.

### Photographic adjustment and proportion generation

**Fig. 3:**
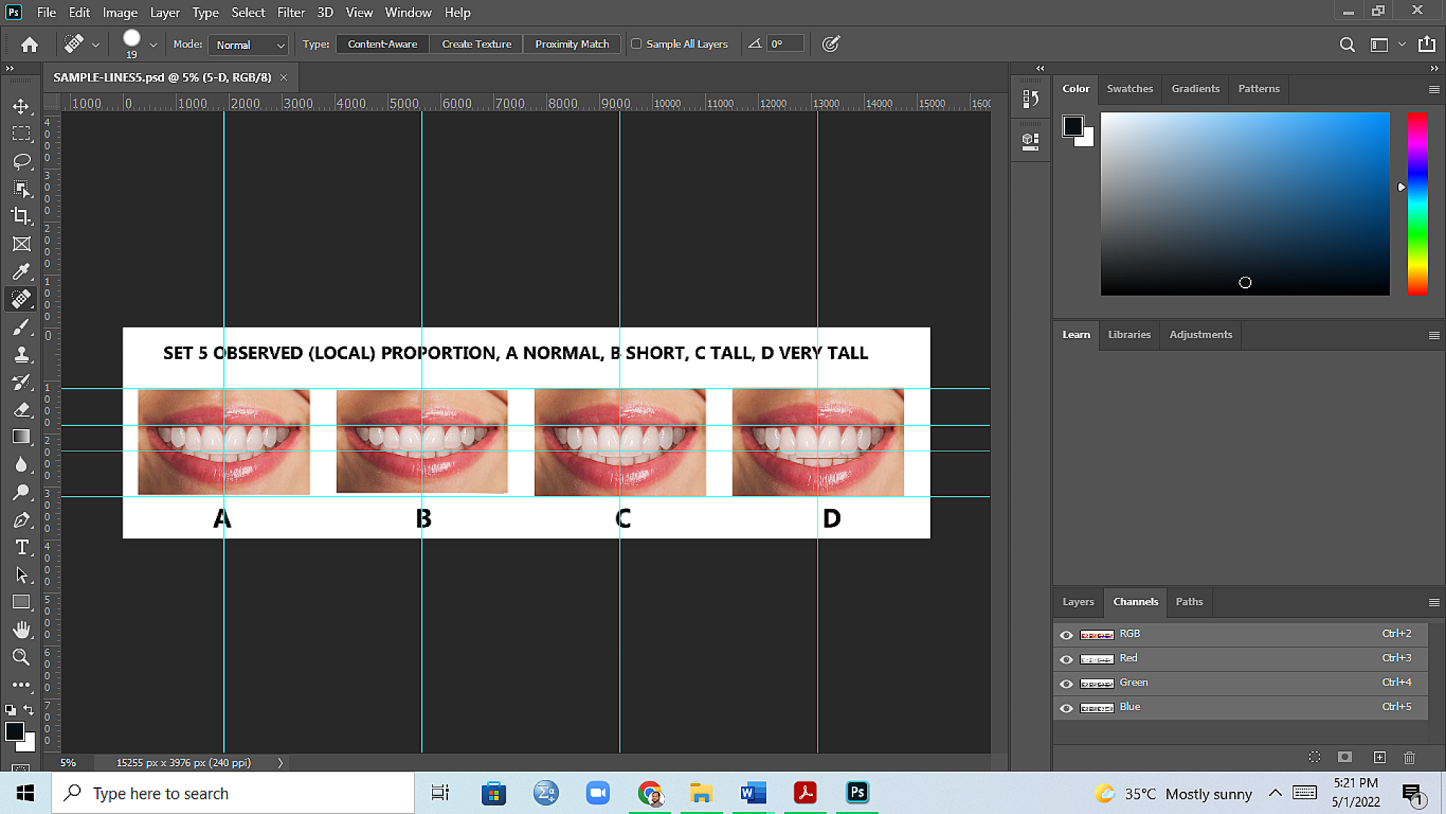
Processing of reference smile images in adobe photoshop software

The digital image was modified using computer software (Adobe, version 21.0.2, San Jose, CA, United States) to create a symmetrical left-to-right tooth that included a length of central incisor 9.459 mm lateral incisor 8.789 mm and canine 8.822 mm. The width of the central incisor was kept at 8.047 mm, the lateral incisor at 6.112 mm, and the canine tooth at 6.501 mm. The width-to-height ratio of the central incisor teeth was maintained at 85%. Other features included changes made to the teeth and the gingival contours, lip line harmony, tooth display, symmetry, natural appearance, and proportional harmony. The photos were then proportionately warped (scaled) to create teeth with four distinct height and breadth ratios: normal, short, tall, and very tall height teeth. Subsequently, the photographs were cropped to ensure uniform image sizes. The resulting collection of photos was labeled as the local or observed proportion Fig. 3. It is important to note that the manipulation was confined to maxillary anterior teeth only, and neither the posterior nor mandibular anterior teeth were altered.

**Fig. 4:**
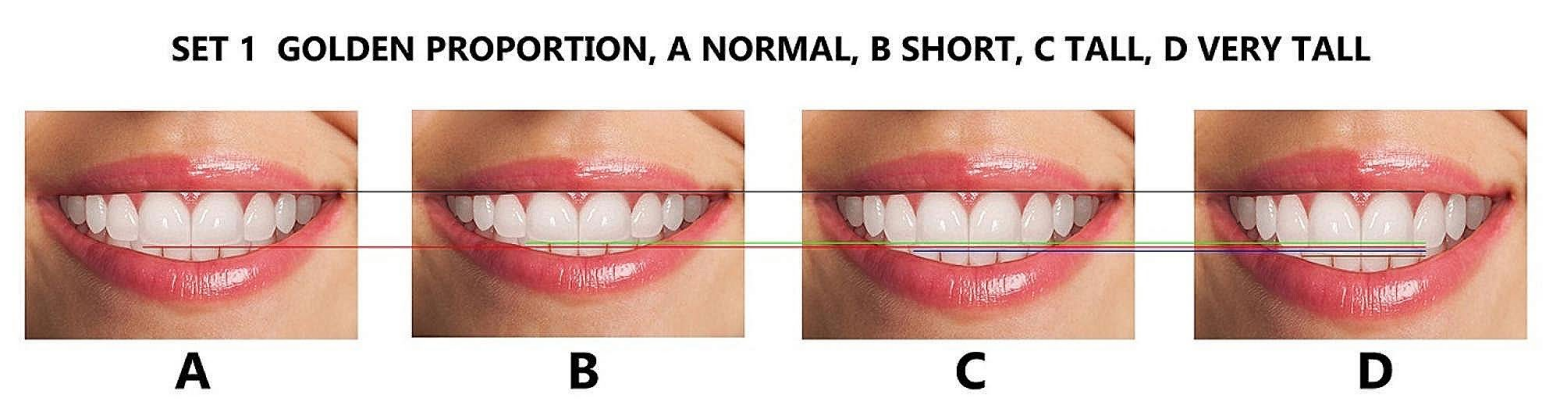
The golden proportion smile set, (**A**); normal height, (**B**): short, (**C**); tall, (**D**); very tall. Image B is 1 mm smaller than A, image C is 1 mm larger than A, and image D is 2 mm larger than A in height

**Fig. 5:**
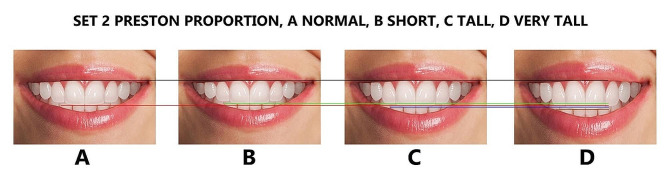
The Preston proportion smile set, (**A**); normal height, (**B**): short, (**C**); tall, (**D**); very tall. Image B is 1 mm smaller than A, image C is 1 mm larger than A, and image D is 2 mm larger than A in height

**Fig. 6:**
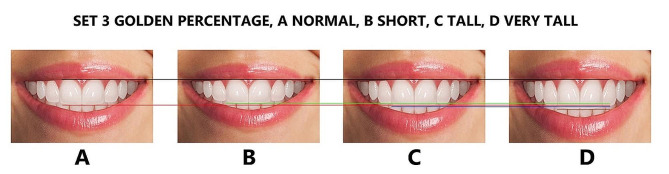
The golden percentage smile set is (**A**), normal height; (**B**), short; (**C**), tall; and (**D**), very tall. Image B is 1 mm smaller than A, image C is 1 mm larger than A, and image D is 2 mm larger than A in height

**Fig. 7:**
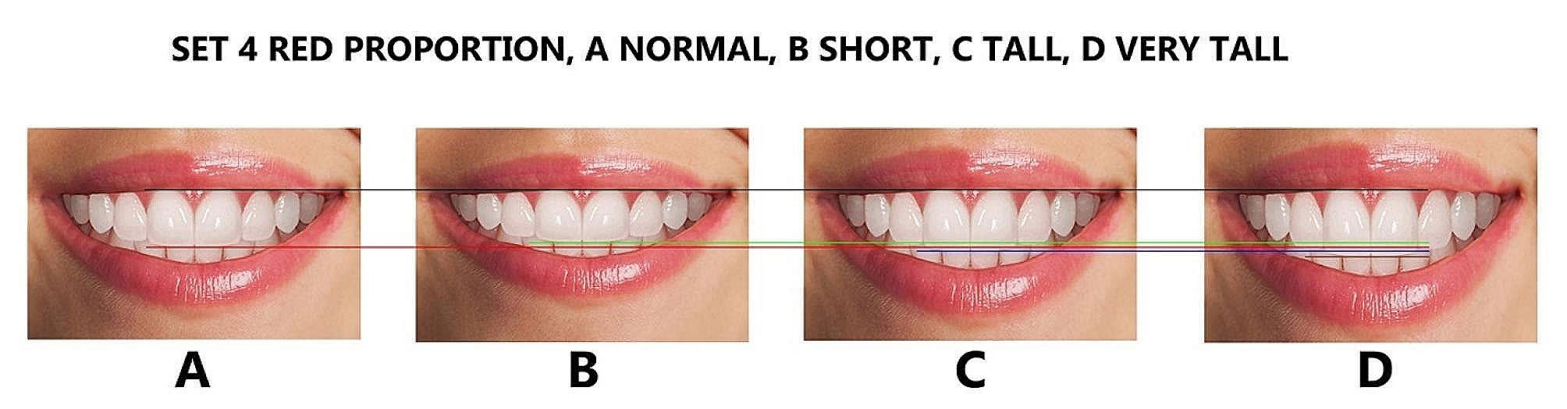
The RED proportion smile set, (**A**); normal height, (**B**): short, (**C**); tall, (**D**); very tall. Image B is 1 mm smaller than A, image C is 1 mm larger than A, and image D is 2 mm larger than A in height

**Fig. 8:**
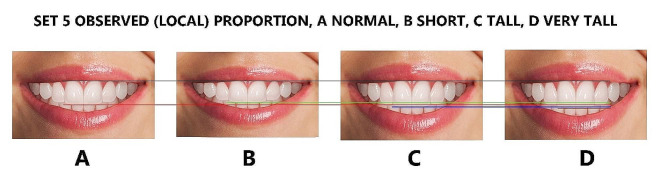
The observed smile set, (**A**); normal height, (**B**): short, (**C**); tall, (**D**); very tall. Image B is 1 mm smaller than A, image C is 1 mm larger than A, and image D is 2 mm larger than A in height

Finally, the maxillary lateral and canines were adjusted for each height group to generate additional tooth proportions, including golden and RED proportions, golden percentage, and Preston proportion. These proportions were in addition to the local or observed proportions derived from the Pakistani population (Figs. 4, 5, 6, 7 and 8), resulting in 16 photos. The dental proportions, namely GP (golden proportion), RED proportion, GM (golden percentage), and PRP (Preston proportion), were created based on the width of anterior teeth using metrics shown in Table 1. The maxillary canine-to-canine distance was kept constant in each photograph. Each tooth’s mesiodistal width and incisogingival height were measured using the imaging software’s measurement tool, as well as the width-to-height ratios and anterior contact areas.

**Table 1:** Distribution of anterior teeth width determination metrics

Dental proportions	Central incisor (CI MDW)	Lateral incisor (LI MDW)	Canine (C MDW)
RED proportion	ITCD/ 2(1 + RED + RED2)	CI×0.70	LI×0.70
Golden proportion	ITCD × 0.25	CI ×0.62	LI×0.62
Preston proportion	ITCD/ 2(1.52)*	CI ×0.66	LI ×0.84
Golden percentage	ITCD × 0.25	ITCD × 0.15	ITCD × 0.10

CI: central incisor, LI: lateral incisor, C: canine, MDW: mesiodistal width, ITCD: inter-canine width, ^*^ 1.5 = 0.66 + 0.84, RED: recurring esthetic dental proportion

In all sets, the images were labeled from A to D. Image B was 1 mm smaller in height cervicoincisally than A, while image C was 1 mm larger than A, and image D was 2 mm larger than A in height. The height groups were randomly sequenced, as were the four proportions in each group. The images and ranking field forms were displayed on a laptop screen for dentists to evaluate attractive smiles by viewing them directly.

### Image presentation and dentist assessment

The images were precisely aligned, with a slight adjustment in the position of the lips so that only the affected teeth would appear to move. This was done to make the selection more definitive with the fewest distractions. The same laptop computer (Dell Inspiron 5000 series, Dell, Inc., Round Rock, TX, USA) was used throughout the survey to ensure identical viewing times. A total of 111 general dental practitioners and 33 specialists were positioned in front of the screen one by one within the confines of its width to reduce angular distortion. A set of initial instructions was projected, and a demonstration set of two differently proportioned smiles (not subsequently displayed in the survey) was shown to familiarize the viewers with the protocol that would follow.

Each view was displayed for 3 s and then faded to the other view for 3 s, repeated four times. Subsequently, each view was shown for 1 s for three more times. This sequence was maintained throughout the survey. Participants were asked if there were any questions. Once the program began, nothing further was said, and participants were instructed to remain silent. Each view in the set smoothly faded to the other view seven times, with a 5-second interval between each set to allow time for recording their responses on the survey form. After the five sets of smiles had been shown, participants were prompted on the screen to identify the primary proportion that influenced their decisions. The protocol used was similar to the one described by (W.H. Ward, 2007) [15].

For a total of 20 responses, dentists were asked to score each of the four photographs in each group from good to bad. A questionnaire (Supplementary Form 1) was employed to collect demographic data, including name, age, gender, level of education, occupation, email address, if any, part of a residence in Pakistan, postal code address, year of graduation from dental school, the dental school graduated from, principal professional activity, general dentist, or specialist, and the number of anterior teeth restored. Responses to the various fields were gathered and saved in a separate folder for future review.

All the photographs and required teeth measurements, including the dentists’ responses, were carried out by one investigator (N.A.), except for the interoperator assessment stage, where another operator (R.J.) assisted in avoiding data collection errors.

### Statistical analysis

The collected data was entered into the statistical package for social science software (SPSS Version 24.0; Chicago, IL, USA). Descriptive analysis of continuous and categorical variables was performed. Qualitative variables like gender and smile perception were calculated for mean and standard deviation—quantitative variables like age and responses of dentists and specialists.

The distribution of data was analyzed using normality plots and Shapiro-Wilk. For the decision of dentists on attractive smile perception, one sample t-test and paired t-test were used to determine respondents’ opinions of the good and bad tooth proportion and width/height ratio from the five choices, Golden proportion, RED proportion, golden percentage, Preston proportion, and local or observed proportion values of Pakistani citizens. The Chi-square test evaluated gender differences and factors influencing smile attractiveness. A *p*-value of ≤ 0.05 was considered to be statistically significant.

## Results

The demographic data of dentists are presented in Table 2. The study included 143 participants. The mean age of participants was 32.468 ± 7.917, the minimum age recorded was 21 years, while the maximum age of participants noted in this study was 57 years. The age range of participants included in this study was 36.00 years. Participants had varied experience levels, with 52.44% having 0–5 years, 27.27% with 6–10 years, and 4.89% with 11–15 years. Additionally, 46.3% were general dentists, 13.8% were specialists. Regarding patient load in the last 60 days, 48.25% treated 0–9 patients, 21.67% treated 10–19 patients, and 11.88% treated 20–29 patients. A small percentage treated more than 30 patients (7.69%), while 10.48% did not report. The mean number of patients treated was 25.63 (± 1.28).

**Table 2:** Demographic data of dental practitioners (*n* = 143)

Demographic	N	%
Sex		
Male	71	49.65
Female	72	50.34
Total	143	100
Age of participants		
Mean and SD	32.468 ± 7.917	
Experience in years		
0 to 5	75	52.44
6 to 10	39	27.27
11 to 15	7	4.89
16 to 20	13	9.09
More than 21	9	6.29
Total	143	100
Mean and SD	28.6 ± 1.85	
Professional activity		
General Dentist	111	46.3
Specialist	33	13.8
Total	143	100
Number of patients treated in last 60 days		
00–09	69	48.25
10–19	31	21.67
20–29	17	11.88
More than 30	11	7.69
Not reported	15	10.48
Total	128	100
Mean and SD	25.63 ± 1.281	

SD: standard deviation

In this study, the apparent and actual width of maxillary anterior teeth revealed significant differences. For the right central incisor, right lateral incisor, and right canine, the perceived widths (8.130, 6.241, 6.619, millimeters (mm) were smaller than the actual widths (8.627, 7.371, 7.864 mm), with p-values of 0.061, 0.071, and 0.063 mm respectively. Similarly, for the left central incisor, left lateral incisor, and left canine, perceived widths (7.965, 5.983, and 6.384 mm) were smaller than actual widths (8.723, 7.623, 7.959 mm), with p-values of 0.075, 0.014, and 0.027, respectively. Combining the widths of all six teeth also showed a significant difference (*p* = 0.001), with the perceived width (40.788 mm) being smaller than the actual width (48.170 mm), as shown in Table 3.

**Table 3:** The distribution of mean maxillary anterior teeth width (*n* = 230)

Maxillary teeth	Actual width		Perceived width		p-value
	Mean (mm)	Standard deviation	Mean (mm)	Standard deviation	
Right central incisor	8.627	0.453	8.130	0.717	0.061
Right lateral incisor	7.371	0.539	6.241	0.903	0.071
Right Canine	7.864	0.457	6.619	1.319	0.063
Left central incisor	8.723	0.479	7.965	0.848	0.075
Left lateral incisor	7.623	0.637	5.983	0.937	0.014
Left canine	7.959	0.482	6.384	1.320	0.027
Combine six teeth width	48.170	1.551	40.788	4.090	0.001

The mean length of the maxillary right central incisor was 9.990 ± 0.883. In contrast, the lateral incisor was 9.093 ± 0.642. The length of a canine tooth was 8.805 ± 0.638. However, on the left side of the arch, the mean length of the central incisor tooth was 8.929 ± 0.520, and the lateral incisor had a mean length of 8.485 ± 0.691. In comparison, the mean length of the canine tooth was 8.839 ± 0.884. The mean length of the central incisor was 9.459 ± 0.701, the lateral incisor 8.789 ± 0.666, and the canine 8.822 ± 0.761. The width-to-height ratio of the right and left central incisor teeth was 81.381 ± 1.329 and 89.203 ± 9.688, respectively. The mean width-to-height ratio recorded was 85.305 ± 8.798.

### The preference of smile attractiveness by dentists

Participants’ smile preferences were evaluated using three broad categories: good, fair, and bad for normal, small, tall, and extra-tall teeth. The majority of participants preferred the RED proportion as an attractive smile. The breakdown of categories is as follows: for normal-sized anterior teeth, 83 (58.04%) participants rated it as “good,” 42 (29.37%) as “fair,” and 18 (12.58%) as a “bad” smile. In the case of small-sized teeth, only 1 (0.69%) participant considered the RED proportion as “good,” 9 (6.29%) as “fair,” while the majority, 133 (93%), chose it as a “bad” smile. Similar results were observed for tall and very tall teeth. The preference for the RED proportion significantly differed across different tooth sizes (*p* < 0.001).

Furthermore, dentists selected the golden proportion-based smile as the second-best option. For normal-sized teeth, it was rated as “good” by 71 (49.65%) dentists, “fair” by 32 (22.37%), and categorized as “bad” by 40 (27.97%) dentists. However, more than two-thirds of dentists rated it as “bad” for small to very tall teeth. Dentists’ preferences differed significantly for normal, tiny, tall, and very tall teeth, with 62% preferring the golden proportion (*p* = 0.001).

The Preston proportion was rated as “good” by 38 (26.57%) dentists, “fair” by 52 (36.36%), and categorized as “bad” by 53 (27.06%). However, most dentists rated the PRP smile as “bad” for tiny, tall, and very tall teeth. Dentists’ preferences for various tooth sizes differed significantly (*p* = 0.001).

Finally, for normal-sized teeth, the local proportion was evaluated as “good” by 23 (16.08%) dentists, “fair” by 23 (16.08%), and “bad” by 97 (67.83%). Furthermore, more than three-quarters of dentists rated tiny, tall, and very tall teeth based on the local proportion as “bad.” As presented in Table 4, there was a significant difference (*p* = 0.001) between dentists’ preferences for the local proportion when evaluated in normal size and small to tall teeth.

**Table 4:** Comparison of dentists’ preferences within constructed smile groups (*n* = 143)

Smile Preference	Group (A) Golden proportion				p-value
	Normal N%	Small N%	Tall N%	Very tall N%	
Good	71 (49.65)	6 (4.19)	27 (18.88)	3 (2.09)	0.001
Fair	32 (22.37)	5 (3.49)	9 (6.29)	5 (3.49)	
Bad	40 (27.97)	138 (96.50)	107 (74.82)	135 (94.40)	
Total	143 (100)	143 (100)	143 (100)	143 (100)	
Group (B) Recurring esthetic dental proportion					
Good	83 (58.04)	1 (0.69)	4 (2.79)	3 (2.09)	0.001
Fair	42 (29.37)	9 (6.29)	7 (4.89)	4 (2.79)	
Bad	18 (12.58)	133 (93)	132 (92.30)	136 (95.10)	
Total	143 (100)	143 (100)	143 (100)	143 (100)	
Group (C) Golden percentage					
Good	55 (38.46)	7 (4.89)	3 (2.09)	2 (1.39)	0.001
Fair	30 (20.97)	5 (3.49)	4 (2.79)	6 (4.19)	
Bad	58 (40.55)	131 (91.60)	136 (95.10)	135 (94.40)	
Total	143 (100)	132 (100)	143 (100)	143 (100)	
Group (D) Preston proportion					
Good	38 (26.57)	4 (2.79)	4 (2.79)	5 (3.49)	0.001
Fair	52 (36.36)	6 (4.19)	7 (4.89)	4 (2.79)	
Bad	53 (37.06)	133 (93)	132 (92.30)	134 (93.70)	
Total	143 (100)	143 (100)	143 (100)	143 (100)	
Group (E) Local proportion					
Good	23 (16.08)	11 (7.69)	2 (1.39)	2 (1.39)	0.001
Fair	23 (16.08)	5 (3.49)	5 (3.49)	3 (2.09)	
Bad	97 (67.83)	127 (88.81)	136 (95.10)	138 (96.50)	
Total	143 (100)	143 (100)	143 (100)	143 (100)	

Group A: golden proportion, Group B: recurrent esthetic dental proportion, Group C: golden percentage, Group D: Preston proportion, Group D: Local/observed proportion: The proportion ratio between the width of anterior teeth obtained in this study was used to construct a smile with different teeth length, P value of ≤ 0.05 was considered statistically significant

Table 5 presents a comparison of dentist’s preferences for various smiles. Dentists’ perceptions of the golden and RED proportions in normal-sized teeth differed significantly (*p* = 0.001), indicating that dentists favored RED proportion-based smiles over golden proportion-based smiles. Similarly, a significant difference (*p* = 0.001) was observed between the two dental proportions in tall teeth, with dentists rating the golden proportion-based smile as superior to the RED proportion. However, there was no significant difference (p˃0.05) between very tall and small teeth.

**Table 5:** Comparison between the perceptions of dentists regarding the constructed dental smile (*n* = 143)

Dental Proportion groups	Tooth size	Level of preference p-value
Group A and B	Normal	0.001
	Small	0.071
	Tall	0.001
	Very tall	0.276
Group A and C	Normal	0.001
	Small	0.159
	Tall	0.002
	Very tall	0.675
Group A and D	Normal	0.001
	Small	0.091
	Tall	0.005
	Very tall	0.449
Group A and E	Normal	0.001
	Small	0.074
	Tall	0.026
	Very tall	0.085
Group B and C	Normal	0.001
	Small	0.794
	Tall	0.371
	Very tall	0.905
Group B and D	Normal	0.001
	Small	0.063
	Tall	0.153
	Very tall	0.214
Group B and E	Normal	0.001
	Small	0.071
	Tall	0.391
	Very tall	0.249
Group C and D	Normal	0.014
	Small	0.361
	Tall	0.259
	Very tall	0.291
Group C and E	Normal	0.001
	Small	0.392
	Tall	0.073
	Very tall	0.062
Group D and E	Normal	0.002
	Small	0.471
	Tall	0.612
	Very tall	0.743

Group A: golden proportion, Group B: recurrent esthetic dental proportion, Group C: golden percentage, Group D: Preston proportion, Group E: Local proportion: The proportion ratio between the width of anterior teeth obtained in this study was used to construct a smile with different teeth length, a p-value of ≤ 0.05 was considered statistically significant

Furthermore, dentists observed a significant difference when a golden proportion-based smile was compared to a golden percentage-based smile in normal-sized teeth (*p* = 0.001). The dentists preferred a smile that was based more on the golden proportion. Similarly, there was a significant difference (*p* = 0.002) in tall teeth, with dentists preferring golden proportion over golden percentage. In contrast, there was no significant difference (p˃0.05) in smile perception between very tall and small teeth.

Moreover, when the golden proportion-based created smile was compared to the Preston proportion in normal-sized teeth, a significant difference (*p* = 0.001) was observed. Dentists favored golden proportion smiles over Preston proportion smiles. Regarding tall-sized teeth, a significant difference (*p* = 0.005) was found between the golden proportion and the Preston proportion-based smile. There was no statistically significant difference (p˃0.05) between very tall size and small teeth constructed with golden proportion and Preston proportion.

Additionally, the dentist’s choice for an appealing smile differed considerably (*p* = 0.001) between a smile made in golden proportion and one created in local proportion in normal-sized teeth. Dentists preferred the golden proportion to the local proportion-based smile. There was a significant difference when tall teeth were compared across dentists (*p* = 0.026). The participants favored a golden proportion-based smile over a local proportion-based smile. There was no significant difference (p˃0.05) between golden proportion and local proportion-based smiles in small and very tall teeth.

A significant difference (*p* = 0.001) was found between the dentist’s preference for smiles built with RED proportion and the golden percentage in normal-sized teeth. Dentists favored a RED proportion-based smile over a golden percentage-based smile. When the two dental proportions were used to produce a smile, no significant difference (p˃0.05) was seen in small, tall, and very tall teeth.

There was a significant difference (*p* = 0.001) in dentist preference between the RED proportion and the Preston proportion-based smile in normal-sized teeth, whereas no significant difference was noted between dentists’ choices for small, tall, and very tall-sized teeth. Furthermore, dentists found a considerable difference when comparing RED proportion with local proportion-based smiles in normal-sized teeth (*p* = 0.001). The dentists chose the RED proportion above the local proportion. At the same time, there was no significant difference (p˃0.05) between the two dental proportions when small, tall, and very tall teeth were compared. The dentist’s judgment of the smile formed with the golden percentage and Preston proportion was substantially different (*p* = 0.014) in normal-sized teeth. Dentists favored the golden percentage above the Preston proportion. Apart from that, no significant difference (p˃0.05) in preference was detected when the two dental proportions were compared in small, tall, and very tall teeth.

Furthermore, in normal-sized teeth, there was a significant difference (*p* = 0.001) between the preference for the golden percentage and local proportion-based smiles. The golden percentage was selected by dentists above the local proportion. There was no difference in dental preference between small, tall, and very tall teeth. Lastly, a significant difference (*p* = 0.002) was detected between the dentist’s viewpoint in normal teeth when the Preston proportion was compared to the local proportion. When opinions on the two dental proportions in tiny, tall, and very tall teeth were compared, no significant difference (p˃0.05) was detected.

### Comparison of constructed smile preference by dentists and specialists

Regarding RED proportion in normal teeth, the comparison of smile choice between general dentists and specialists was as follows: 68 general dentists picked it as good, 25 were fair, and 18 preferred RED proportion-based constructed smiles as bad. While 16 professional specialist dentists chose it as a good smile, 17 chose it as a fair smile, and 0 chose it as a bad smile. Only one general dentist thought the RED proportion in little teeth was good, five thought it was fair, and 105 thought it was bad. The specialists did not choose a good smile; however, 4 chose a fair smile, and the bulk of 29 chose a bad one. Furthermore, none of the general dentists rated it as a good smile, three chose it as fair, and most 108 chose it as a bad-formed smile. Four specialists chose a good smile, four chose a fair smile, and 25 chose a bad smile. Finally, in very tall teeth, two general dentists rated the smile as good, two as fair, and 107 as bad. The RED proportion was rated as good and fair by 0 specialists, with the majority of 33 calling it a poorly built smile.

The golden proportion-based created smile in normal teeth was rated as good by 58 general dentists, fair by 30, and bad by 23. According to the specialist dentists, the golden proportion, a good smile with regular teeth, was preferred by 13 participants. Three thought it was a good smile, while 17 thought it was a bad smile. Six general dentists thought it was good, five thought it was fair, and the rest of the 100 dentists thought it was bad. In the instance of teeth, two specialists chose a good smile, one chose a fair smile, and thirty chose a bad smile. Furthermore, 23 general dentists classified the golden proportion as a good smile in tall teeth, whereas 5 classified it as a fair smile, and 83 as a bad smile. Similarly, four specialists thought tall teeth were good, four thought fair teeth were good, and 25 considered it a bad smile. Additionally, one general dentist preferred a good smile with very tall-sized teeth, 5 preferred a fair smile, and the majority of 105 general dentists preferred a bad smile. However, in the case of very tall teeth, all 33 specialists rated a smile built on golden proportions as bad.

The comparison of smile choice between general dentists and specialists in the case of golden percentage-based created smile in normal teeth was as follows: 49 general dentists picked it as good, 17 fair, and 45 preferred golden percentage-based constructed smile as bad. Six expert specialist dentists rated it as good, 13 as fair, and 14 as terrible. Only five general dentists rated small teeth as good, four as acceptable, and 102 as bad. One specialist dentist chose a good smile, no dentists chose a fair smile, and the majority of 32 chose a bad smile. Furthermore, with tall teeth, three general dentists chose a good smile, four chose a fair smile, and the majority of 104 chose a bad-formed smile. No specialist preferred a good or fair smile, whereas 33 preferred it as a bad smile. Furthermore, in very tall teeth, one general dentist rated the smile as good, two as fair, and 108 as bad. The golden percentage was rated as good by one specialist, fair by 0 specialists, and bad by 32.

The Preston proportion-based created smile in normal teeth was rated good by 25 general dentists, fair by 37, and bad by 49. The Preston procedure was preferred by specialist dentists, with 14 participants achieving a nice smile with natural teeth. Meanwhile, 15 considered it a fair smile, and 4 preferred it as a bad smile. Small-size teeth were rated as good by 0 general dentists, fair by 5, and bad by 106 dentists. Regarding smiling teeth, four specialists chose a good smile, two chose a fair smile, and 27 chose a bad smile. Furthermore, 0 general dentists classified the Preston proportion as a good smile in tall teeth, 3 classified it as a fair smile, and 108 classified it as a bad smile. Similarly, four specialists thought tall teeth were good, five thought it was fair, and 24 thought it was bad. Furthermore, three general dentists preferred a good smile in very tall-sized teeth, two preferred a fair smile, and the majority of 106 general dentists preferred it as a bad smile. On the other hand, the RED proportion was evaluated as good and fair by two specialists each, while the bulk of 29 picked it as a bad smile.

The comparison of smile choice between general dentists and specialists in the instance of local proportion in normal teeth was as follows: 19 general dentists picked it as good, 20 as fair, and 72 as bad. While four specialized dentists chose it as a good smile, three chose it as a fair smile, and 26 chose it as a bad smile. The local proportion of little teeth was rated as good by 0 general dentists, fair by 1, and bad by 97. One specialized dentist chose a good smile, one dentist chose a fair smile, and 31 dentists chose a bad smile. Furthermore, with tall teeth, two general dentists chose a good smile, four chose a fair smile, and the majority of 105 chose a bad-formed smile. No specialist preferred it as a good or fair smile, whereas 33 preferred it as a bad smile. Regarding very tall teeth, two general dentists rated the smile as good, one as fair, and 108 as bad. The local percentage was rated as good by two specialists, fair by two others, and bad by the vast majority of 29.

The most prevalent element influencing dentists’ smile preferences was teeth arrangement (137 dentists), followed by tooth color (107 dentists) and midline (83 dentists). The fourth key aspect, as chosen by 77 dentists, was the composition of teeth. About 105 dentists chose the central incisor tooth as the most essential tooth in the smile. The least preferred factor was the gingival zenith, which only 13 dentists chose. Only ten dentists chose the lateral incisor teeth as their least preferred tooth.

There was a significant difference between dentists in terms of gingival display (*p* = 0.001), TA (*p* = 0.011), MDL (*p* = 0.003), EMB (*p* = 0.017), GZ (*p* = 0.005), and ACA (*p* = 0.038). However, there was no difference in dentists’ preferences in TC (*p* = 0.590) or SL (*p* = 0.847).

Furthermore, both male and female dentists identified tooth arrangement as the most influential factor influencing the dental smile. Similarly, 47 male and 58 female dentists chose the central incisor as the most significant tooth in dental smiles. The variables influencing smile preference differed between male and female dentists, with a substantial difference reported in GD, TA, MDL, COT, CI, Ca SA, SL, and ACA (*p* = 0.05). But there was no significant change in LI (*p* = 0.212), EMB (*p* = 0.135), or GZ (*p* = 0.366), as presented in Table 6.

**Table 6:** Distribution of the factors affecting smile preference in dentists of both sexes

Variables %		GD	TA	MDL	COT	TC	CI	LI	CA	TS	EMB	SA	SL	GZ	ACA
Dentist	Yes	92	137	83	107	77	105	10	33	42	26	43	49	13	62
	No	51	6	60	35	66	38	133	110	101	117	100	94	130	81
p-value		0.001	0.011	0.003	0.001	0.590	0.001	0.015	0.017	0.001	0.017	0.016	0.847	0.005	0.038
Male	Yes	42	69	48	59	39	47	7	19	27	22	21	39	9	29
	No	29	2	23	12	32	24	64	52	44	49	50	32	62	42
Female	Yes	50	68	35	48	38	58	3	14	15	4	22	10	4	33
	No	22	4	37	23	34	14	69	58	57	68	50	62	68	39
p-value		0.001	0.001	0.001	0.001	0.001	0.001	0.212	0.001	0.001	0.135	0.002	0.001	0.366	0.001

GD: gingival display, TA: teeth arrangement, MDL: midline, COT: the color of teeth, TC: teeth composition, CI: central incisor, LI: lateral incisor, Ca: Canine, TS: tooth size, EMB: embrasure, SA: smile arc, SL: smile line, GZ: gingival zenith, ACA: anterior contact area, p–value ≤ 0.05 was considered significant (Chi-square analysis)

## Discussion

The selection of different tooth proportions to create aesthetic smiles varies from dentist to dentist, primarily due to their perception of what is considered an aesthetic smile. Furthermore, tooth proportions vary in different ethnicities as maxillary teeth’ width-to-height ratio varies. According to the results of this study, most of the dentists preferred the RED proportion for teeth of normal size as they considered it to be. These findings correspond with a study by Rosenstiel SF [9], where it was concluded that for normal-sized teeth, the RED proportion was the preferred tooth proportion. However, dentists sometimes do not consider the RED proportion the best preference for normal-sized teeth, as found in our study. Such results correspond with a study in the literature that states similar findings [15]. In some patients, it has been noticed that the maxillary central incisors appear taller, wider, and more visible when smiling compared to other maxillary anterior teeth. This might explain why dentists prefer the golden over the RED proportion. Moreover, the RED proportion is not frequently found in the patient’s natural dentition, as described by Shetty et al. [16].

In our study, participants rated the golden proportion as the second-best option for normal-sized teeth, receiving a “Good” rating in terms of smile perception. However, for small to very tall teeth, the majority of dentists considered the golden proportion as a less favorable choice. Existing literature indicates that the presence of the golden proportion varies among populations, with some studies confirming its existence [7, 17]. Contrarily, our investigation into the local Pakistani population did not reveal a prevalent golden proportion [18]. This aligns with the notion that the golden proportion might not be consistently present across diverse populations. A clinical study even reported that only 17% of the lateral-central incisor ratio correlated with the golden proportion, and there was a reduced likelihood of finding a canine-lateral incisor ratio of 0.618 [16]. Despite the infrequency of the golden proportion in certain populations, it is noteworthy that some dentists still prefer it over other tooth proportions [9].

In our study, some dentists selected Preston’s proportion as the preferred option for normal-sized teeth. Such conclusions correspond with a study in the literature that states similar findings as for normal-sized teeth, the Preston proportion was preferred over the golden proportion [19]. However, in our research, most dentists considered preston proportion unsuitable for small, tall, and very tall teeth. These results are similar to a study by Rosenthal where it was reported that golden proportion was the preferred choice for short, very short, and very tall teeth [9]. However, such findings are not always found; variations among ethnicities and genders are always present. Furthermore, in a study by Krishna P Lashkari [20], Preston proportion was found among the central and lateral incisors in all of the study’s female participants. This further helps us understand that variations in tooth proportions exist amongst different genders.

In our study, when comparing tooth proportions in different constructed smiles, dentists favored the RED proportion-based smile over the golden proportion for normal-sized teeth. This finding aligns with a study by Ward, where most dentists expressed a preference for the RED proportion over the golden proportion, particularly for teeth of normal length [15, 21]. Conversely, for tall teeth in our study, the majority of dentists supported the golden proportion rather than the RED proportion. In the context of normal-sized teeth, 75 to 80% crown width height ratio, when comparing the golden and Preston proportions, dentists in our study favored the golden proportion. However, it is essential to note that numerous studies have reported that smiles constructed with golden proportions may not always result in aesthetic appeal. Instead, the racial and individual features of a smile should be given preference when creating a dental smile [22, 23].

Moreover, in our study, the participants preferred the golden proportion over the local proportion in the population. Amongst the comparison of normal-sized tooth proportions between RED proportion and golden percentage, the dentists of our study opted for the RED proportion-based smile. However, a survey by Azimi and colleagues concluded that the RED proportion cannot be used as a constant proportion to create aesthetic smiles in patients [24]. Furthermore, the golden percentage was preferred by the dentists of our study over the Preston proportion for normal-sized teeth. A study by Kalia and colleagues found that the Preston proportion holds little value in terms of aesthetic dentistry as it does not represent natural aesthetic smiles, but a modified golden percentage is more useful for smile design [25].

Since specialist dentists receive more training in advanced dentistry than general dental practitioners, a difference in preference for tooth proportions is found. The specialists and general dentists agreed to normal-sized teeth as they considered the RED proportion suitable. A study by Saha et al. concluded that general dentists had a larger number of smiles and were found to be more agreeable than specialists as they received advanced training and knowledge. Hence, they were more critical of the construction of smiles [26]. Furthermore, rather than focusing entirely on tooth proportion, a smile constructed while keeping the facial aesthetic in mind can also be proven beneficial for the overall creation of an aesthetic smile for the patients. For small teeth, the specialists and general dentists considered the RED proportion a bad option. About the golden proportion for small teeth, most general dentists considered it to be a good option; however, specialists regarded it as a bad option. A study by Gillen et al. concluded that a poor correlation exists between golden proportion and tooth dimensions in terms of the construction of aesthetic smiles [27]. Additionally, general dentists and specialists considered golden proportions a bad option for tall teeth. Considering the additional training and knowledge of the specialists, this makes them more critical and demanding in terms of aesthetic smiles.

Regarding the golden percentage, most general dentists and specialists prefer not to use this proportion to construct aesthetic smiles. Furthermore, concerning Preston’s proportion, conflicting results were obtained between general dentists’ and specialists’ preferences according to different tooth sizes. Such findings can be due to extra consideration that the specialists provide in terms of aesthetics and function as compared to the general dentists. Moreover, general dentists and specialists considered local proportions a bad option for constructing an aesthetic smile. These results could be due to local proportions failing to consider facial and dental aesthetics.

In our study, the factors that the dentists most commonly noted for smile construction were the arrangement of the teeth, the color of the teeth, and the midline. These findings correspond with a study by Egle Ong and colleagues [28], who concluded that teeth arrangement and color are important factors to consider when constructing an aesthetic smile. The teeth arrangement and color of the teeth are also regarded as important by the patients themselves, as the color and arrangement of teeth are the first thing that they notice while smiling. Moreover, in terms of the importance of teeth, while smiling, the dentists considered central incisors as the most important teeth in our study. Such results correspond with the study of Fahad F Alsulaimani [29], which emphasizes the importance of central incisors as the most important teeth while smiling.

Furthermore, the number of cases treated by dentists in the study emerges as a crucial determinant influencing the overall results. Among the 143 participants, a breakdown of patient loads in the last 60 days reveals that 48.25% treated 0–9 patients, 21.67% treated 10–19 patients, and 11.88% treated 20–29 patients. Notably, 7.69% managed more than 30 patients, while 10.48% did not report their patient load. This diverse distribution highlights the varying clinical experiences among participants. Dentists handling a substantial caseload, exemplified by the 48.25% treating 0–9 patients, likely bring extensive clinical expertise to their evaluations of smile characteristics. The study’s findings, particularly regarding preferences for different tooth proportions, are intricately linked to the diverse patient loads and experiences of participating dentists. The distribution of dentists across different experience levels and patient load categories adds a quantitative layer to understanding their professional backgrounds, which may contribute to the observed trends in smile preferences.

The study displays notable strengths, including its diverse participant sample involving general dentists and specialists, providing a comprehensive perspective on tooth proportion preferences. Its relevance to the local Pakistani population enhances the applicability of the findings to the cultural and ethnic characteristics of this specific group. Furthermore, the study’s thorough exploration of multiple tooth proportions, encompassing RED proportion, golden proportion, golden percentage, and Preston proportion, contributes to a comprehensive analysis of aesthetic preferences. However, certain limitations should be acknowledged, including potential minor inaccuracies in measuring dental casts that may impact result precision. While our study provides valuable insights into dentist preferences for attractive smiles in the Pakistani population, we recognize that the sample may not fully represent the entire dental community. To enhance the generalizability of our findings, future research could involve a larger and more diverse sample, including dentists from various regions and practice settings. This expansion would contribute to a more comprehensive understanding of the broader perspectives within the dental profession in Pakistan. Additionally, the subjectivity inherent in smile perception, especially among dental professionals, may not fully capture the diversity of perspectives in the general population. While valuable, the study was focused on tooth proportions, leaving out other important factors contributing to an aesthetic smile, such as lip architecture, facial proportions, and soft tissue landmarks.

The study findings suggest several recommendations for future research and clinical practices in aesthetic dentistry. Further in-depth cultural analyses are warranted to understand the influences of cultural diversity on smile preferences, allowing for more tailored treatments across diverse populations. Long-term follow-up studies should be conducted to assess the stability and satisfaction of patients with different tooth proportions over an extended period, providing insights into the longevity of aesthetic interventions. Emphasizing clear communication between dental practitioners and patients regarding aesthetic preferences is crucial, and implementing advanced digital technologies, such as artificial intelligence and virtual reality, in smile design processes should be explored. Ongoing professional development for dental practitioners, focusing on the latest digital smile design tools and techniques, is essential to ensure proficiency in incorporating technological advancements into clinical workflows. Prioritizing research on patient-centered outcomes, interdisciplinary collaboration, public awareness campaigns, and ethical considerations will contribute to holistic and ethical care in aesthetic dentistry.

## Conclusions

The smiles created using the principles of RED proportion were opted as the most attractive by Pakistani dentists. Tooth arrangement, the color of teeth, and the midline are considered other important factors for constructing an aesthetic smile.

### Electronic supplementary material

Below is the link to the electronic supplementary material.

Supplementary Material 1

## Data Availability

The data presented in this study will be available on request from the corresponding author.
