# Supplementary material for: Smile aesthetics in Pakistani population: dentist preferences and perceptions of anterior teeth proportion and harmony
Source: BMC Oral Health. 2024 Mar 29;24:401. doi: 10.1186/s12903-024-04100-4 (PMC10979575; doi:10.1186/s12903-024-04100-4)
Supplement: Supplementary file 1 — Supplementary Material 1 [file 12903_2024_4100_MOESM1_ESM.docx]

**Supplementary form 1**

**Questionnaire for the perception of smile attractiveness in the Pakistani population**

Form number: ­­­­­­­­­­­­­­­­­________________

Date: _______________________

Age: ________________________

Gender: _________________ ____

Postal code address: ___________________________________________________

E-mail address if any: ____________________________________________________________________

Occupation: _________________________________________________________

Level of education: ___________________________________________________

Year of graduation from dental school: ____________________________________

Principal professional activity:

General dentist Specialist

The number of patients treated in the last 60 days in which the proportion, crown width height ratio of the 6 maxillary anterior teeth were altered:________________

Comments if any please: ____________________________________________________________________

**Likert like scale**

For each of the questions below, circle the response that best characterizes how you perceive the dental smile attractiveness in 5 sets of pictures shown to you, where 1 = bad, 2 = fair, 3 = good.

| Comment on smile in each set | A  Normal or average | | | B  Short teeth | | | | C  Tall teeth | | | D  Very tall teeth | | |
| --- | --- | --- | --- | --- | --- | --- | --- | --- | --- | --- | --- | --- | --- |
|  |  |  |  |  |  |  |  |  |  |  |  |  |  |
| Set 1 | 1 | 2 | 3 | | 1 | 2 | 3 | 1 | 2 | 3 | 1 | 2 | 3 |
| Set 2 | 1 | 2 | 3 | | 1 | 2 | 3 | 1 | 2 | 3 | 1 | 2 | 3 |
| Set 3 | 1 | 2 | 3 | | 1 | 2 | 3 | 1 | 2 | 3 | 1 | 2 | 3 |
| Set 4 | 1 | 2 | 3 | | 1 | 2 | 3 | 1 | 2 | 3 | 1 | 2 | 3 |
| Set 5 | 1 | 2 | 3 | | 1 | 2 | 3 | 1 | 2 | 3 | 1 | 2 | 3 |

Set 1: golden proportion; Set 2: Preston proportion; Set 3: golden percentage; Set 4: recurring esthetic dental proportion; Set 5: local / observed proportion
